# Amaranth’s 2-Caffeoylisocitric Acid—An Anti-Inflammatory Caffeic Acid Derivative That Impairs NF-κB Signaling in LPS-Challenged RAW 264.7 Macrophages

**DOI:** 10.3390/nu11030571

**Published:** 2019-03-07

**Authors:** David Schröter, Susanne Neugart, Monika Schreiner, Tilman Grune, Sascha Rohn, Christiane Ott

**Affiliations:** 1 Leibniz Institute of Vegetable and Ornamental Crops e.V. (IGZ), 14979 Grossbeeren, Germany; Schroeter@igzev.de (D.S.); Neugart@igzev.de (S.N.); Schreiner@igzev.de (M.S.); 2 Department of Molecular Toxicology, German Institute of Human Nutrition Potsdam-Rehbruecke (DIfE), 14558 Nuthetal, Germany; scientific.director@dife.de; 3 Institute of Food Chemistry, Hamburg School of Food Science, University of Hamburg, 20146 Hamburg, Germany; Rohn@chemie.uni-hamburg.de; 4 Institute of Nutrition, University of Potsdam, 14558 Nuthetal, Germany

**Keywords:** inflammation, caffeic acid derivatives, RAW 264.7 macrophages, NF-κB, amaranth

## Abstract

For centuries, *Amaranthus* sp. were used as food, ornamentals, and medication. Molecular mechanisms, explaining the health beneficial properties of amaranth, are not yet understood, but have been attributed to secondary metabolites, such as phenolic compounds. One of the most abundant phenolic compounds in amaranth leaves is 2-caffeoylisocitric acid (C-IA) and regarding food occurrence, C-IA is exclusively found in various amaranth species. In the present study, the anti-inflammatory activity of C-IA, chlorogenic acid, and caffeic acid in LPS-challenged macrophages (RAW 264.7) has been investigated and cellular contents of the caffeic acid derivatives (CADs) were quantified in the cells and media. The CADs were quantified in the cell lysates in nanomolar concentrations, indicating a cellular uptake. Treatment of LPS-challenged RAW 264.7 cells with 10 µM of CADs counteracted the LPS effects and led to significantly lower mRNA and protein levels of inducible nitric oxide synthase, tumor necrosis factor alpha, and interleukin 6, by directly decreasing the translocation of the nuclear factor κB/Rel-like containing protein 65 into the nucleus. This work provides new insights into the molecular mechanisms that attribute to amaranth’s anti-inflammatory properties and highlights C-IA’s potential as a health-beneficial compound for future research.

## 1. Introduction

In ancient times, the Aztecs were the first to cultivate *Amaranthus* sp. for food production, as ornamental and medicinal plants [1]. Since then, the genus has been distributed globally, comprising at least 70 species [2]. In Europe and America, amaranth seeds (mainly from *A. hypochondriacus*, *A. cruentus,* and *A. caudatus*) are used as pseudocereals (i.e., utilized like cereals of the Poaceae family). However, in parts of Africa and Asia, amaranth leaves are an integral part of the diet (mainly from *A. tricolor*, *A. hybridus*, and *A. dubios*) [1,3]. Leaves of vegetable amaranth are used as potherb for the preparation of soups or stews, cooked or fried with other green vegetables as a side or main dish, or the young leaves are eaten raw in salads [1,2]. Furthermore, they have been used in various traditional remedies, e.g., Ayurvedic medicine, for the treatment of inflammation [1]. However, it remains unclear how amaranth-derived preparations facilitate anti-inflammatory action on a molecular basis.

Health beneficial properties of plant-derived medicinal preparations are most commonly attributed to plant secondary metabolites, for example, phenolic acids. Amaranth leaves contain high amounts of phenolic acids, especially hydroxycinnamic acid derivatives and among these, most abundantly, 2-caffeoylisocitric acid (C-IA) [4]. Chlorogenic acid (3-caffeoylquinic acid, CQA), a related compound that is not found in amaranth leaves, but is widely distributed in other plants, also contains caffeic acid (CA) as a phenolic moiety. CQA has been shown to possess numerous biological properties, including antioxidant, anti-inflammatory, and anti-carcinogenic activities [5,6,7,8,9]. Previous studies showed that the biotransformation of CQA and C-IA by the gut microbiome is comparable and that both compounds can be partially resorbed by the human organism [10,11,12]. However, the health beneficial properties of C-IA have not yet been studied. Besides, substantial efforts have been made to find compounds that regulate and modulate the activation of inflammatory cells. The most thoroughly investigated caffeic acid derivative in this aspect is the caffeic acid phenylethyl ester (CAPE). This compound occurs in various plants and is also a constituent of propolis [13]. It has been shown to be a potent inhibitor of NF-κB activation, reducing the extent of inflammation. The potential drawback of CAPE is that higher concentrations can lead to interferences with other cellular processes, which might counteract the protective activity [14,15,16].

To figure out whether a natural compound has anti-inflammatory activity, cellular studies are often performed with cultivated RAW 264.7 macrophages. Macrophages are cells that are considered as a defense system of the organism upon infections. Macrophages are recruited by signaling molecules, such as interferon γ (INFγ) and tumor necrosis factor alpha (TNFα), as well as lipopolysaccharide (LPS) from the cell wall of gram-negative bacteria, to phagocytose pathogens and infected cells. Subsequently, macrophages release cytokines (e.g., interleukin 1 & 6 (IL-1 & 6) and TNFα) and inflammatory mediators (e.g., nitric oxide) to activate other immune cells [17]. LPS-mediated activation of macrophages stimulates the translation of the inducible nitric oxide synthase (iNOS) catalyzing the production of nitric oxide as an immune defense mechanism and as signaling molecules. This process is carried out by various intracellular signaling cascades that trigger the translocation of transcription factors such as the nuclear factor κB family (NF-κB) into the nucleus [18]. The mammalian NF-κB superfamily comprises many members, including RelA (p65), NF-κB1 (p50; p105), NF-κB2 (p52; p100), c-Rel, and RelB. Each of the NF-κB members, except RelB, can form homo- and heterodimers with one another to facilitate several cellular responses, including the regulation of proliferation, apoptosis, and the inflammatory response. The main activated form in inflammatory processes is a heterodimer of the p65 subunit comprising p50 or p52. These NF-κB complexes occur in the cytosol in an inactive form resulting from their association with inhibitory IκBα proteins. Upon, e.g., inflammation, phosphorylation of IkBα by IKKs leads to the dissociation of the inhibitory complex. The NF-κB subunit then translocates into the nucleus to activate the transcription of κB target genes, while the inhibitory IκBα subunit is ubiquitinated and undergoes proteasomal degradation [19]. The resulting expression of cytokines and inflammatory mediators, and their subsequent release into the extracellular matrix, facilitates the recruitment of other immune cells. Therefore, excessive activation of immune cells leads to immunopathological effects on the surrounding tissue. Numerous diseases, such as cardiovascular diseases, atherosclerosis, or even cancer, have an inflammatory component, accompanied by an overreacting immune system [20,21,22].

This study aimed to show whether amaranth’s C-IA possesses an immune response regulating activity in physiologically plausible concentrations. Specifically, anti-inflammatory effects of C-IA in comparison to CA and CQA were investigated in a cellular system. Thus, RAW 264.7 macrophages were incubated with selected caffeic acid derivatives (CADs) to study (1) the uptake by the cells and the recovery of the CADs and (2) the anti-inflammatory potential of CADs. Therefore, cell experiments were carried out to show the CADs effect on iNOS induction by observing iNOS mRNA and protein levels, as well as NO release from the cell into the media. Furthermore, we investigated (3) the effect of CADs treatment on the nuclear translocation of p65 and (4) how CADs modulate the expression levels of the NF-κB target genes TNFα and IL-6.

## 2. Materials and Methods

### 2.1. Chemicals and Cell Culture Reagents

Water (MS-grade) was purchased from VWR. ACN (MS-grade), and caffeic acid, chlorogenic acid, and CAPE were obtained from Sigma Aldrich. RPMI 1640 (with stable glutamine, 2.0 g·L^−1^ NaHCO_3_) cell culture medium was purchased from Biochrom (FG 1215).

### 2.2. Isolation of the C-IA from Amaranth Leaves

Isolation of the C-IA from the leaves of amaranth was carried out as previously described [4], with some minor changes. Here, leaves of *Amaranthus hybridus* cv. Kongei and cv. IP-7 were used. Briefly, 500 mg of freeze-dried plant material was stirred for 30 min with 8 mL of 60% MeOH and centrifuged for 5 min at 986× *g*. The supernatant was decanted and evaporated to dryness. The aqueous extracts were then loaded on a preconditioned (2 mL of MeOH, then 2 mL of 0.1% formic acid) SPE-cartridge (Chromabond^®^ C18ec, 3 mL/500 mg Macherey-Nagel GmbH & Co. KG). The column was washed with 2 mL of 0.1% formic acid. Then, the phenolic compounds were eluted with a mixture of acidic water and acetonitrile (0.1% formic acid/70% water/30% ACN). The eluate was evaporated to a tenth of its original volume. The C-IA was isolated using preparative liquid chromatography and the purity was evaluated as already described in Schröter et al. [4]. However, instead of 0.5% acetic acid, 0.1% formic acid was applied in Solvent A. Purity (HPLC-DAD 320 nm) of the isolated C-IA was 91%. Contaminations were due to other isomers of the caffeoylisocitric acid ester in the isolates. Besides, no further contaminants were detected by either HPLC-DAD or HPLC-ESI-MS detection.

### 2.3. Cell Culture and Treatment

Cell experiments were performed with the murine macrophage cell line RAW 264.7. Macrophages were cultured using RPMI 1640 medium supplemented with 10% fetal calf serum (FCS) in an atmosphere of 5% CO_2_ and 37 °C. Cells were passaged using Trypsin-EDTA and experiments were done within three to four passages. CADs were solved in 50% EtOH and were diluted with sterile-filtered water in concentrations from 1–50 µM and 0.1% of EtOH before application to the cell culture medium. For the C-IA treatments, the isolated compound from amaranth leaves was used. CA, CQA, and CAPE treatments were carried out using commercially available compounds.

### 2.4. Cell Viability

To evaluate cytotoxicity, 3.5 × 10^4^ cells were seeded into each well of a 96-well plate with 200 µL of cell culture medium. After 24 h, supernatants were removed and 200 µL of treatment solution was added in three different cavities to perform triplicate analysis within the assay. Treatment solutions were composed of a defined concentration (1–50 µM) of the CADs and 0.1% of ethanol, the solvent control consisted of 0.1% ethanol, and the control was only cell culture medium. Cells were incubated for a total of 24 h with the different treatment solutions. Afterwards, supernatants were removed and MTT or Neutral-Red solutions were added and incubated for 1 h. After solubilization of the colorants with solubilization solution (10% sodium dodecyl sulfate (SDS)/1.2% acetic acid/88.8% water) and distaining solution (1% acetic acid/50% water/49.9% EtOH), respectively, the optical density (OD) was measured at 580 nm and 540 nm, respectively. OD values obtained were normalized by setting the control values to 100% and values of assay triplicates were averaged. MTT and Neutral-Red experiments were carried out in triplicate.

### 2.5. Quantification of the CADs Concentrations in the Supernatants and Cells

To quantify the concentrations of the different CADs, 2 × 10^6^ cells were seeded in 10 mL cell culture medium in a petri dish with a diameter of 10 cm. After 24 h of attachment and proliferation, the cells reached a semi-confluent status. The cell culture medium was removed and the CADs where diluted in fresh cell culture medium for 10 µM treatment solutions. The cells where then incubated with 5 mL of the treatment solutions for 4 and 24 h. Control cells were incubated with 0.1% of EtOH. After incubation, supernatants were collected, 200 µL vitamin C-EDTA (Vc-EDTA) solution was added (400 mM Na_2_HPO_4_, 0.1% EDTA, 10% l-(+)-ascorbic acid), and samples were frozen immediately at −80 °C. Afterwards, cells were washed twice with 5 mL of PBS and 500 µL SDS-lysis buffer (10 mM Tris-HCl, pH 7.5, 0.9% Nonidet P-40, 0.1% SDS and 1 mM Pefabloc) was added. Lysates where then collected, and protein concentrations were determined using the Lowry assay (manufacturer’s instructions) The excess lysates were mixed with 20 µL of Vc-EDTA solution and frozen at −80 °C prior to analysis. To analyze the concentrations in the supernatants and cell lysates, 500 µL of the solutions was thawed and mixed with an enzyme solution (500 U/mL β-glucuronidase and 40 U/mL aryl sulfatase). Solutions were incubated at 37 °C under continuous shaking for 1 h and then loaded on a pre-conditioned SPE-cartridge and treated as described above. Collected eluates were mixed with 10 µL 1% l-(+)-ascorbic acid solution and evaporated to dryness. The resulting pellets were dissolved in 250 µL 50% ACN. Cell lysate eluates were then measured directly with HPLC-MS/MS, while the eluates of the supernatants were diluted 1:100 with 50% acetonitrile prior to analysis. HPLC-MS/MS analyses were carried out with the chromatographic methods described in Volmer et al. [10], with minor modifications: For each compound, the optimal declustering potential, entrance potential, collisions energy, collision exit potential, and characteristic Multiple Reaction Monitoring (MRM) transitions were determined (Supplementary Table S1). For the quantification, the MRM transition with the highest intensity and lowest variation within five injections was used. MRM transitions with a lower intensity were used as qualifiers. The method included CA, CQA, and C-IA. An external calibration was used for quantification and data analysis was carried out with Analyst^®^ 1.6.2 software from Sciex Germany GmbH. All the samples were done in triplicate. The CAD concentrations in the supernatant were calculated with respect to the initially applied 5 mL of cell medium. The obtained values for the cell lysates from the HPLC-MS/MS measurement were normalized to protein concentrations based on the Lowry assay, because the protein content represents a reliable basis for the quantification of the CAD concentrations in the lysates:

$$c_{lysates}\left[\frac{nmol}{mg\;protein}\right]=\;\frac{\frac{nmol}{mL}\;\left(HPLC-MS\right)}{\frac{mg}{mL\;}protein\;\left(Lowry\right)}$$

### 2.6. iNOS Protein Concentrations and NO Production

To assess the iNOS protein concentrations, 2.5 × 10^5^ cells were seeded in a six-well plate with 2 mL of cell culture medium. After 24 h, the medium was removed and either 10 µM solutions of the CADs, 0.1% ethanol solution, and/or LPS (1 µg/mL) were added to the wells. For the solvent control, a 0.1% ethanol solution was applied. For the LPS treatment, 1 µg/mL LPS was added. The control was only incubated with cell culture medium. After 24 h of treatment, the supernatants were collected and immediately frozen at −20 °C prior to NO analysis. After duplicate washing with PBS, cells were lysed with 100 µL of SDS lysis buffer, scratched from the surface, and left on ice for 30 min. Afterwards, lysates were repeatedly disrupted through a 27G syringe and centrifuged at 20,000× *g* for 10 min at 4 °C. Protein concentrations in the supernatants were obtained by the Lowry protein assay (manufacturer instructions). Reducing Laemmli buffer (0.25 M Tris, pH 6.8, 8% SDS, 40% glycerol, 0.03% orange G) was added to 25 µg protein of each sample and proteins were denatured at 95 °C for 5 min. Samples were separated by SDS-PAGE and transferred to a nitrocellulose membrane. Blocking was performed using Odysseys Blocking Buffer (LICOR; 927-40000) 1:5 diluted in PBS. Primary and secondary antibodies were also diluted in Odysseys Blocking Buffer/PBS, containing 0.1% Tween-20. Anti-iNOS rabbit (Novus Biologicals; NBP1-50606) was used as the primary antibody. The iNOS protein values obtained were normalized to GAPDH using anti-GAPDH mouse (Abcam; ab8245). The experiments were carried out in triplicate, setting the control to 100%.

NO concentrations in the supernatants were assessed following the protocol of the nitric oxide assay kit “Griess Reagent System” (Promega; G2930). Obtained values were divided by the protein concentrations for normalization. The experiments were done in triplicate.

### 2.7. NF-κB/RelA p65 Translocation

To study the nuclear translocation of NF-κB/RelA (p65), 2 × 10^6^ cells were seeded in 10 mL cell culture medium in a petri dish with a diameter of 10 cm. After 24 h, cell culture medium was removed and CADs (10 µM), EtOH (0.1%) and/or LPS (1 µg/mL) were added for 4 h. Cells were then washed twice with 5 mL of PBS. Afterwards, 5 mL of PBS was added twice for collecting and transferring the cells into a centrifugation tube. After gentle shaking, an aliquot of 1 mL was taken and centrifuged at 250× *g* for 5 min at 4 °C and the remaining pellet was dried and frozen at −80 °C prior to qPCR experiments. The residual 9 mL was centrifuged at 250× *g* for 5 min at 4 °C. The supernatant was discarded, and the remaining pellet was suspended in 400 µL hypotonic homogenization buffer (HHB) (20 mM HEPES, 1 mM EDTA, proteinase inhibitor 1:1000 (Sigma; P8340), phosphatase inhibitor 1:500 (100 mM NaVaO4), pH 7.5). A potter homogenizer was used on ice for 150 repetitions. Homogenates were transferred to a 2-mL tube on ice and then centrifuged at 750× *g* for 15 min at 4 °C. The cytosolic supernatant was transferred into a new tube and the nuclear pellet was washed once with 200 µL HHB and centrifuged at 750× *g* for 15 min at 4 °C. The cytosolic supernatant was centrifuged at 20,000× *g* for 15 min at 4 °C and the supernatants were collected. The washed nuclear pellet was suspended in 100 µL HBB and homogenized by 10 ultra-sonic shocks. The suspension was then centrifuged at 20,000× *g* for 15 min at 4 °C and the supernatants were collected. Protein concentrations were obtained by the Bradford protein assay and samples were prepared further according to the description above. An anti-p65 rabbit antibody (Cell Signaling Technology; D15E12) was used as the primary antibody for the p65 detection. For both nuclear and cytosolic fractions, the Coomassie stained membrane was used to normalize the obtained values.

### 2.8. PCR Analysis

The mRNA from the cell pellets was extracted using the dynabeads^®^ mRNA purification kit (Life Technologies; 61012), following the provided protocol.

The cDNA synthesis was carried out following the protocol for the SensiFAST™ cDNA Synthesis Kit (Bioline; BIO-65053). In brief, 15 µL of mRNA solution, 4 µL of TransAmp buffer, and 1 µL reverse transcriptase were mixed gently by pipetting. The thermocycler was set up as follows: 10 min 25 °C, 15 min 42 °C, 5 min 85 °C, holding 4 °C. Obtained cDNA solutions were diluted with 80 µL of 10 mM Tris-HCl with 0.1 mM EDTA with pH 8 and stored at −20 °C prior to analysis.

The q-PCR was carried out using the Dream-Taq-HS-polymerase protocol (Thermo Fisher Scientific; 15619374). Briefly, 11 µL of sterile water was added to each well of a 96-well plate, followed by 1 µL of template (cDNA). For each well, 13 µL of master mix (912.5 µL sterile water, 250 µL 10-fold buffer, 50 µL dNTPs, 25 µL forward primer (25 µM), 25 µL reverse primer (25 µM), 25 µL 10-fold SYBR Green and 12.5 µL Taq-polymerase) was added. Table 1 provides the primers used for the different experiments. Amplicons were generated by applying an initial denaturation temperature of 95 °C for 5 min, followed by 40 cycles of 95 °C (for 15 s), 60 °C (30 s), and 72 °C (30 s), as well as a melting curve measurement cycle of 95 °C (30 s), 60 °C (30 s), and 95 °C (30 s). The results obtained for the amplicons of GAPDH and HPRT were combined and used as a normalization factor.

### 2.9. ELISA TNFα and IL-6 Protein Concentration

To assess the TNFα concentration supernatants of the iNOS protein western blot preparations (24 h incubation time) were used. The quantification was done following the protocol of the Invitrogen™ TNF alpha Mouse Uncoated ELISA Kit (88-7324-22).

Quantification of IL-6 was carried out with the supernatants of the iNOS protein western blot preparations (24 h incubation time) following the protocol of the Mouse IL-6 Quantikine ELISA Kit (M6000B) from R&D Systems™. Obtained values from the two ELISA kits were normalized to the protein concentrations of the individual samples.

### 2.10. Statistical Analysis

Statistical analyses were performed using Prism 5 software (GraphPad). All experiments were done as independent triplicates (using cells with different passage numbers) and data are presented as mean ± SD. A one-way ANOVA Tukey’s post-hoc test was used. *p* values of ≤0.05 were accepted as statistically significant differences.

## 3. Results

In this study, the anti-inflammatory effects of selected plant-derived CADs on LPS-challenged murine RAW 264.7 macrophages were evaluated. Each of the selected compounds contains a CA moiety in its chemical structure, as depicted in Table 2. Among these compounds, CA and CQA are widespread in plants, whereas C-IA has only been described for the genera *Amaranthus* and *Dactylus*. Nevertheless, in amaranth species, C-IA concentrations are comparable to CA and CQA concentrations of other vegetables. Based on the literature emphasizing the absorption of CADs and assuming a 200 g portion of vegetables, circulation contents of CADs might range between 4–70 µM (Table 2).

None of the CADs showed reduced cell viability in either the Neutral-Red (Supplementary Figure S1A) or the MTT-assay (Supplementary Figure S1B) in concentrations up to 50 µM, besides CAPE, which reached the IC_70_ above 10 µM. Following this result, an application of 10 µM of each CAD was found to be an optimum concentration for further experiments.

### 3.1. Cellular Uptake and Stability of CADs in Cell Culture Medium

**Figure 1:**
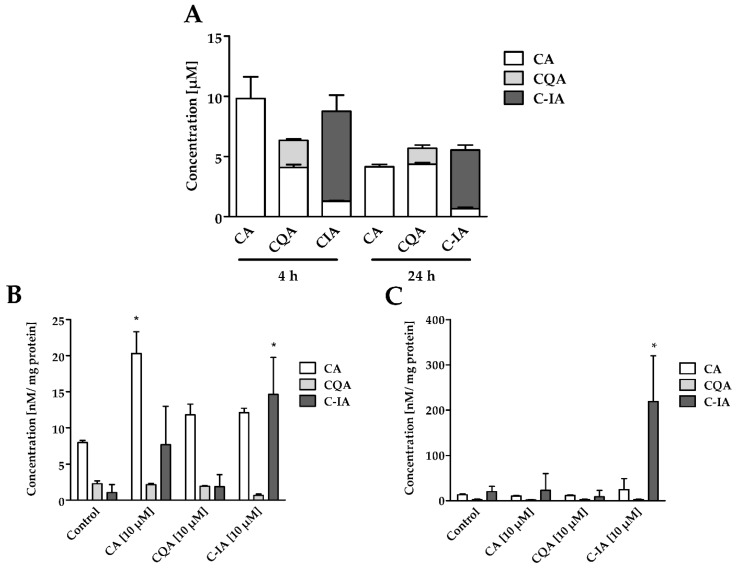
(**A**) Concentrations of CADs in the RAW264.7 cell supernatants after 4 h and 24 h incubation with 10 µM solutions and a basal concentration of CA, CQA, and C-IA in cell culture medium (RPMI 1640) (*n* = 3); (**B**) Lysate concentrations of CA, CQA, and C-IA in whole lysates of RAW264.7 cells after 4 h incubation with 10 µM solutions and the control (0.1% EtOH) (*n* = 3); (**C**) Lysate concentrations of CA, CQA, and C-IA in whole lysates of RAW264.7 macrophages after 24 h incubation with 10 µM solutions and the control (0.1% EtOH) (*n* = 3); Statistically significant differences derived from one-way ANOVA Tukey’s post-hoc test are indicated by * (*p* ≤ 0.05 vs. control).

To study whether CADs are stable in the cell culture medium and within the time of incubation, RAW 264.7 macrophages were incubated with 10 µM solutions of CA, CQA, and C-IA. Quantification was carried out using two to three compound-specific MRM transitions (Supplementary Table S1). After 4 h incubation of the selected compounds with cell culture medium, 9.82 ± 1.79 µM of the applied CA (almost 100 mol%) and 7.47 ± 1.34 µM (75 mol%) of the original C-IA were found in the supernatants (Figure 1A). For CQA, only 2.25 ± 0.12 µM (22.5 mol%) was detected. In the C-IA and CQA samples, however, CA was detected in proportions of 1.28 ± 0.04 µM (13 mol%) and 4.07 ± 0.25 µM (40 mol%), respectively, of the originally applied 10 µM. After 24 h, the concentrations of CADs had approximately halved (t_1/2_ ~20–24 h). The overall concentrations of CADs tended to remain higher in the CQA and C-IA samples after 24 h incubation. Specifically, in the CA treatment, 4.14 ± 0.20 µM (41 mol%) of the applied 10 µM was recovered, while in the CQA samples, 4.35 ± 0.13 µM (43.5 mol%) of CA and 1.32 ± 0.27 µM (13.2 mol%) of CQA were found. In C-IA samples, 4.87 ± 0.42 µM (48.7 mol%) of C-IA and 0.66 ± 0.11 µM (6.6 mol%) of CA were recovered after 24 h incubation.

The CADs uptake by the RAW 264.7 macrophages is shown in Figure 1B,C. The lysate concentrations of CA after 4 h incubation showed a significantly higher amount of CA in the CA treatment, with 2.5-fold higher CA concentrations than the control (Figure 1B). In the CQA and C-IA treatments, CA concentrations tended to higher amounts, with 1.5-fold higher CA concentrations compared to the control. Furthermore, in the C-IA treatment, significantly higher concentrations of C-IA were found compared to the control (14-fold higher than control). After 24 h, it seems that cells had been adapted to the CA and CQA influx, since no significant differences for the CA and CQA concentrations in the different treatments were observable (Figure 1C). However, amaranth’s C-IA accumulated to 15-fold higher concentrations within 24 h compared to 4 h of treatment.

### 3.2. CADs Treatment Inhibits iNOS Activation

**Figure 2:**
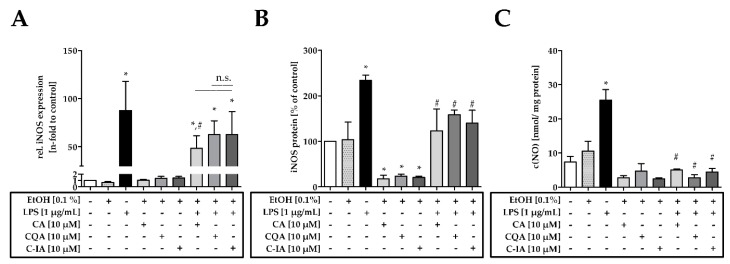
(**A**) iNOS mRNA expression in RAW 264.7 cells after 16 h of incubation with 10 µM CA, CQA, and C-IA solutions (*n* = 3–4); (**B**) iNOS protein levels in RAW 264.7 cells after 24 h incubation with 10 µM CA, CQA, and C-IA solutions (*n* = 3); (**C**) NO concentrations in the supernatants of RAW 264.7 cells in nmol/mg protein after 24 h incubation with 10 µM CA, CQA, and C-IA solutions (*n* = 3); Statistically significant differences derived from one-way ANOVA Tukey’s post-hoc test are indicated by * (*p* ≤ 0.05 vs. control) or # (*p* ≤ 0.05 vs. LPS). Representative blots are depicted in Supplemental Figure S2A.

The anti-inflammatory effects of CADs on the induction of the iNOS were observed at the transcript, protein, and NO production level. On the transcript level, the co-incubation LPS+CA led to a significantly lower number of iNOS transcripts compared to the LPS treatment (Figure 2A). CQA and C-IA co-incubation with LPS showed no significantly different iNOS expression than the LPS+CA or LPS treatment. Western blot analysis for iNOS in the cells after 24 h incubation with CADs showed significantly lower amounts of iNOS protein in the CAD treated cells compared to the control treatment (Figure 2B). Significantly increased iNOS protein levels were found when the cells were challenged with LPS, which were significantly decreased when co-incubating cells with LPS and either CA, CQA, or C-IA. In terms of the NO production level, the co-incubation with CA, CQA, and C-IA led to a reversion of the LPS effect by diminishing the NO concentrations in the supernatants to control levels (Figure 2C).

### 3.3. CADs Modulate Nuclear Translocation of NF-κB/p65 (p65)

**Figure 3:**
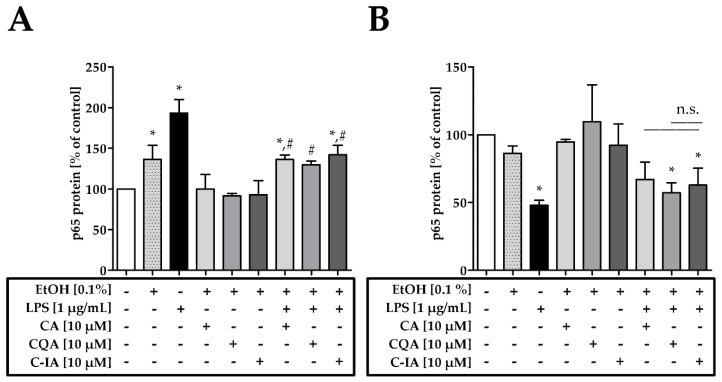
(**A**) p65 protein levels in the nuclear fraction of RAW 264.7 cells after 4 h incubation with 10 µM CA, CQA, and C-IA solutions (*n* = 3); (**B**) p65 protein levels in the cytosolic fraction of RAW 264.7 cells after 4 h incubation with 10 µM CA, CQA, and C-IA solutions (*n* = 4); Statistically significant differences derived from one-way ANOVA Tukey’s post-hoc test are indicated by * (*p* ≤ 0.05 vs. control) or # (*p* ≤ 0.05 vs. LPS). Representative blots are depicted in Supplemental Figure S2B/C.

To understand the reduction of iNOS expression, the translocation of p65 into the nucleus was studied, which targets the transcription of iNOS. Western blot analysis after cellular fractionation showed significantly higher protein levels in the nucleus of LPS-challenged macrophages compared to control treatment (Figure 3A). Co-incubation of LPS with CADs led to a significantly lower p65 translocation compared to the LPS-challenged cells. However, p65 translocation into the nucleus was not reduced to control levels by the co-incubation of LPS with CA, CQA, and C-IA. CADs themselves did not modulate basal p65 translocation. The suppression of the nuclear translocation of p65 by treatment with C-IA was also confirmed via confocal microscopy (Supplemental Figure S3 and Supplemental Method).

For the cytosolic fraction (Figure 3B), it was observed that the LPS treatment led to a significantly lower p65 concentration in the cytosol compared to control. When cells were treated with LPS+CA p65 concentrations were not significantly different to the control. Furthermore, the different CAD treatments were not significantly different to each other.

### 3.4. CADs Modulate TNFα and IL-6 on mRNA Level and Protein

**Figure 4:**
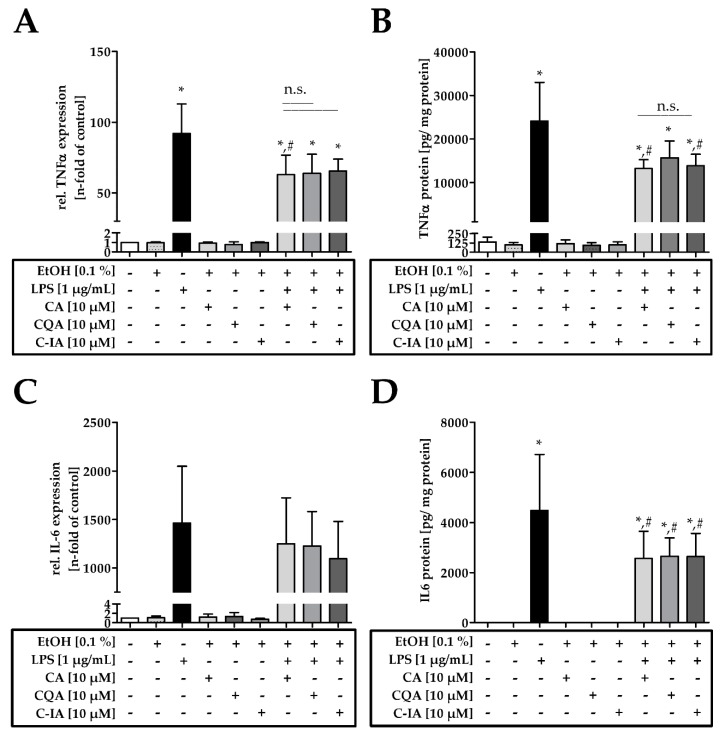
(**A**) TNFα mRNA expression in RAW 264.7 cells after 4 h incubation with 10 µM CA, CQA, and C-IA solutions (*n* = 3–4); (**B**) TNFα protein in the supernatant of RAW 264.7 cells in pg/mg protein after 24 h incubation with 10 µM CA, CQA, and C-IA solutions *n* = 3); (**C**) IL-6 mRNA expression in RAW 264.7 cells after 4 h incubation with 10 µM CA, CQA, and C-IA solutions (*n* = 3); (**D**) IL-6 protein in the supernatant of RAW 264.7 cells after 24 h incubation with 10 µM CA, CQA, and C-IA solutions (*n* = 3). Statistically significant differences, derived from one-way ANOVA Tukey’s post-hoc test are indicated by * (*p* ≤ 0.05 vs. control) or # (*p* ≤ 0.05 vs. LPS).

The impact of the suppressed p65 translocation into the nucleus was further investigated by studying TNFα and IL-6 mRNA and protein levels as both represent p65 targets. The mRNA analysis for TNFα (Figure 4A) showed that LPS-challenged cells expressed 90-fold higher TNFα than the control. Cells incubated with LPS+CA expressed significantly lower TNFα than LPS-challenged cells. The LPS+CQA and LPS+C-IA treatments led to a slightly lower amount of TNFα compared to LPS and were not significantly different to the LPS+CA treatment. The quantitative ELISA for TNFα (Figure 4B) showed a significantly higher amount of protein in LPS-challenged cells compared to the control. After 24 h, co-incubation of LPS with CA and C-IA led to a significant lower amount of TNFα in the supernatant compared to LPS treatment. However, TNFα concentrations remained significantly higher than the control. Similar results were obtained for IL-6 mRNA and protein levels (Figure 4C,D). Incubation with LPS led to a significantly higher expression of IL-6 mRNA compared to the control. Co-incubation of LPS with CA, CQA, and C-IA tended to lower the expression of IL-6 mRNA levels compared to LPS (Figure 4C). IL-6 protein concentrations were only detectable in the LPS treatments. While 24 h LPS treatment alone induced significantly higher IL-6 concentrations in the supernatant compared to the control, co-incubation of CA, CQA, and C-IA with LPS showed significantly lower protein levels compared to the LPS challenged cells (Figure 4D).

## 4. Discussion

To explain the molecular mechanisms of medicinal preparations from amaranth with anti-inflammatory properties, the amaranth-derived phenolic acid C-IA was investigated in comparison to CQA and CA. It was shown that these plant-derived phenolic acids possess no cytotoxic effects towards RAW 264.7 cells in concentrations up to 50 µM. Moreover, it was confirmed that CAPE develops cytotoxic effects in concentrations ≥10 µM [14,15,16]. For the investigation of the anti-inflammatory effects of CA, CQA, and C-IA, a concentration of 10 µM was found to be ideal, since these concentrations are physiologically relevant as they are reachable by a 200 g serving of either crisp head lettuce or amaranth [4,9]. Furthermore, CA and C-IA showed recovery rates of 100% and 75% after 4 h incubation, which indicates a decent stability for further experiments. However, for CQA, only 25% of the initially applied substance could be detected after 4 h incubation besides remarkable concentrations of CA. The losses might relate to the sample preparation, because the enzymes used might have cleaved the ester bond in the CQA, or the work flow, which may not have been ideal for the detection of CQA in the cell lysates. Nevertheless, CADs showed a half-life time of about 20–24 h, indicating that the cells encounter these substances over the course of the experimental time. CADs are extensively metabolized by the human body, as described by Olthof et al. [12]. The mean absorption for CQA and other hydroxycinnamic acids was found to be around 33% by Farah et al. [11]. The absorption rate for amaranth’s C-IA is, hence, unknown, because no bioavailability studies have been performed with this compound yet. However, we hypothesized that C-IA would have the same absorption rate as CQA as we found comparable biotransformation rates when incubating with the human gut microbiome as described in Vollmer et al. [10]. The results of this study suggested that CADs enter the cells by passive diffusion, resulting from the lysate concentrations adjusted with the CADs content in the media after 4 h. In this study, CA and C-IA were detected in the macrophage lysates and C-IA accumulated in the lysates within 24 h of treatment. This indicates that either C-IA can be active over a longer period than CA and CQA or that C-IA is not metabolized to the same extent as the other CADs, so that it can accumulate in the cells within the time of treatment.

This study demonstrates that CA, CQA, and C-IA possess comparable effects on inhibiting iNOS induction and NO production. Induction of iNOS by LPS was shown to be significantly suppressed, as measured at the transcript and protein levels when CADs were present in the cell culture media. Furthermore, LPS-induced upregulation of iNOS could be partially reversed by co-incubation with CADs. In addition, LPS-induced NO production was shown to be significantly lower in the CAD treatments. Two factors may be involved: firstly, CADs treatment led to a lower induction of iNOS and, secondly, CADs are known to be NO scavengers binding to free radicals, such as reactive nitrogen and reactive oxygen species [15,23]. These findings are in accordance with the results of Yang et al., Shin et al., and Landmann et al., although the physiological plausible concentrations applied in the present study were at least 10-fold lower than in those studies [23,24,25]. As a molecular basis for the reduced iNOS induction, the present study describes that the nuclear translocation of p65 in RAW 264.7 macrophages was significantly reduced by the incubation with CA, CQA, and C-IA and that the LPS-induced increase of translocation can be reversed by the CADs treatment. These findings are in accordance with observations made by Song et al., who studied the anti-inflammatory effects of CAPE [26]. Natarajan et al. proposed a covalent modification of a critical sulfhydryl group by CAPE that leads to an inhibited binding of the NF-κB subunits to the DNA [14]. In the present study, however, it was also found that the translocation of p65 into the nucleus was suppressed; reflecting a more upstream inhibition of the phosphorylation cascade. Yang et al. described an inhibition of interleukin-1 receptor-associated kinase 4 (IRAK4) phosphorylation as a target for the CADs [25], which could explain the downstream suppression of NF-κB signaling. This is crucial for the induction of the whole p65 signaling pathway as this would also inhibit IKK and IκBα phosphorylation. Shin et al. proposed that the catechol group of CADs was responsible for the radical scavenging activity, thereby connecting the suppressed activation of transcription factors such as NF-κB in human epithelial cells with reduced oxidative stress inside the cells [23]. Incubation of the macrophages with CADs also suppressed TNFα and IL-6 mRNA and protein levels, confirming that suppressed iNOS levels are due to reduced nuclear translocation of p65, as they all represent NF-κB targets. However, other studies suggest that CADs also influence the signal transduction of activator protein 1 (AP-1), nuclear factor of activated T-cells (NFAT), and various members of the mitogen activated protein kinases (MAPK) cascade [27,28,29], which cannot be excluded within the present study and must be emphasized by further studies.

## 5. Conclusions

Finally, this study showed the anti-inflammatory effects of CADs on LPS-challenged RAW 264.7 macrophages by an inhibition of p65 translocation. These findings help to illustrate the basis of the anti-inflammatory effects of amaranth’s C-IA. Furthermore, it could be outlined that the plant-derived CADs show comparable anti-inflammatory properties to each other in RAW 264.7 macrophages and that the C-IA from amaranth might possess additional cellular effects after accumulating in the macrophages. Hence, it remains unclear in which compartment the cells are depositing the excess C-IA and further studies are needed to shed light on the question of whether the accumulation of C-IA in the cells affects other cellular processes. Furthermore, to evaluate the effectiveness of amaranth-based traditional remedies, future research should emphasize the anti-inflammatory effects of amaranth’s C-IA in vivo. With the results provided, an intervention study on anti-inflammatory effects of C-IA or C-IA-rich amaranth can be designed in mouse models of acute and chronic colitis, and later also in humans that suffer from, e.g., colitis, gastritis, or ulcers. Since the current literature describes that these diseases are all driven by inflammatory processes, which also show highly upregulated iNOS protein levels and increased NF-kB signaling, CADs could be used to reduce inflammatory signaling. Providing knowledge about the impact of amaranth-derived medicinal preparations is highly valuable for its consumers and might therewith increase amaranth’s perception as an alternative crop for future nutrition.

## Figures and Tables

**Table 1:** Target gene primers for qPCR.

Target Gene	Primer	Sequence
GAPDH	forward	GGGTGTGAACCACGAGAAAT
	reverse	GTCTTCTGGGTGGCAGTGAT
HPRT	forward	GCAGTCCCAGCGTCGTG
	reverse	GGCCTCCCATCTCCTTCAT
iNOS	forward	CAGCGCTACAACATCCTGGAGG
	reverse	GGACCAGCCAAATCCAGTCTGC
TNFα	forward	CCACGTCGTAGCAAACCACC
	reverse	TACAACCCATCGGCTGGCAC
IL-6	forward	TCTCTGCAAGAGACTTCCATCCA
	reverse	GTCTGTTGGGAGTGGTATCCTCTG

**Table 2:** Comparison of CAD concentrations in selected foods (^a^ crisphead lettuce or ^b^ amaranth) normalized to serving sizes and the respective absorption to the blood stream.

Compound Name (Abbreviation) Molar Mass	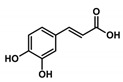 Caffeic Acid (CA) M = 180.16 g/mol	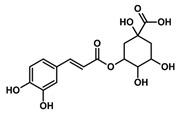 Chlorogenic Acid (CQA) M = 354.31 g/mol	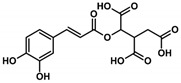 2-Caffeoylisocitric Acid (C-IA) M = 354.27 g/mol
Concentration in vegetables [mg/g FW]	0.15–1.11 [9]	0.13–1.23 [9]	0.20–1.93 [4]
Concentration in vegetables [µmol/g FW]	0.83–6.16	0.37–3.47	0.56–5.45
Content per serving of ^a^ crisphead lettuce or ^b^ amaranth [µmol/200 g portion]	166–1232 ^a^	74–694 ^a^	112–1090 ^b^
Absorbed amount [µmol/200 g portion] (33% mean absorption [11,12])	54.78–406.56	24.42–229.02	36.96–359.70
Concentration in blood stream [µmol/L] (6 L mean Volume)	9.13–67.76	4.07–38.17	6.16–59.95

## Supplementary Materials

The following are available online at https://www.mdpi.com/2072-6643/11/3/571/s1, Figure S1: Cell viability assays, Figure S2: Representative blots, Figure S3: Confocal microscopy of p65 translocation, Table S1: Mass spectrometric parameters, Method: Confocal microscopy of p65 translocation.
