# Supplementary material for: Amaranth’s 2-Caffeoylisocitric Acid—An Anti-Inflammatory Caffeic Acid Derivative That Impairs NF-κB Signaling in LPS-Challenged RAW 264.7 Macrophages
Source: Nutrients. 2019 Mar 7;11(3):571. doi: 10.3390/nu11030571 (PMC6471825; doi:10.3390/nu11030571)
Supplement: Supplementary file 1 [file nutrients-11-00571-s001.zip › Supplemental Method.pdf]

Supplemental Method: Confocal microscopy

To prepare confocal microscopy, 5 x 10^4 cells were seeded in glass bottom dishes with 1 mL of cell culture medium. After 24 h, cell culture medium was removed and C-IAs (10 µM) and/ or LPS (1 µg/mL) were added for 4 h. Cells were then washed twice with 1 mL of PBS. Afterwards, 1 mL of an ice-cold ethanol/ diethyl ether mixture (1:1 v:v) were added and the cells were immobilized for 6 min at -20°C. Then, the cells were washed twice with 1 mL PBS and blocked with 1 mL of an 1% FCS solution in PBS (blocking buffer) for 30 min at room temperature. The cells were washed twice with 1 mL PBS. Then, 80 µL of an anti-p65 rabbit antibody solution (Cell Signaling Technology; D15E12) (1:10 in PBS) were added for the p65 detection and incubated for 2 h at room temperature. The cells were washed twice with 1 mL PBS. An Alexa Fluor 488 goat anti-rabbit antibody (Thermo Fisher Scientific, A-21206) was added and incubated for 30 min at room temperature. Afterwards, the cells were washed twice with 1 mL blocking buffer and then twice with 1 mL PBS. The buffers were removed, and the cells were mounted with a DAPI containing solution (Roth GmBH & Co. KG, HP20.1). Confocal microscopy was carried at with 20x magnification of an LSM 780 (Zeiss). The system was excited at 488 nm (p65, green) and 405 nm (DAPI, blue) to detect p65 and the nucleus, respectively. For each of three independent samples ten pictures with 2-8 cells was taken. The quantification was facilitated by Cyt/Nuc V1.0 Macro via ImageJ described by Grune et al 2018 [1]. Each dot in the scatterplot represents the mean of the p65 ratio in one picture.

[1] Grune, T., Kehm, R., Höhn, A., & Jung, T. (2018). "Cyt/Nuc," a Customizable and Documenting ImageJ Macro for Evaluation of Protein Distributions Between Cytosol and Nucleus. *Biotechnology journal*, *13*(5), 1700652.
