# Supplementary figures and images for: Amaranth’s 2-Caffeoylisocitric Acid—An Anti-Inflammatory Caffeic Acid Derivative That Impairs NF-κB Signaling in LPS-Challenged RAW 264.7 Macrophages
Source: Nutrients. 2019 Mar 7;11(3):571. doi: 10.3390/nu11030571 (PMC6471825; doi:10.3390/nu11030571)

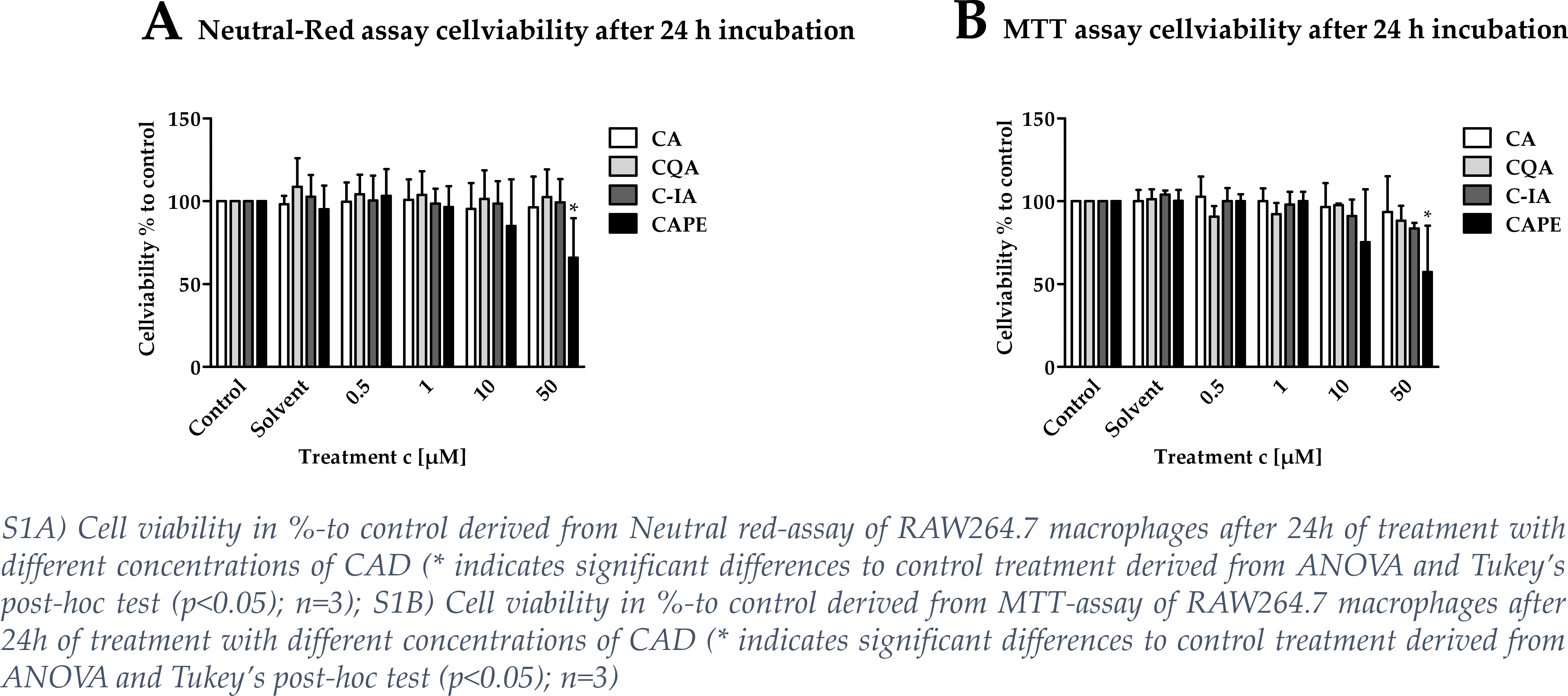

Supplement: Supplementary file 1 [file nutrients-11-00571-s001.zip › Supplemental Figure 1.tif]

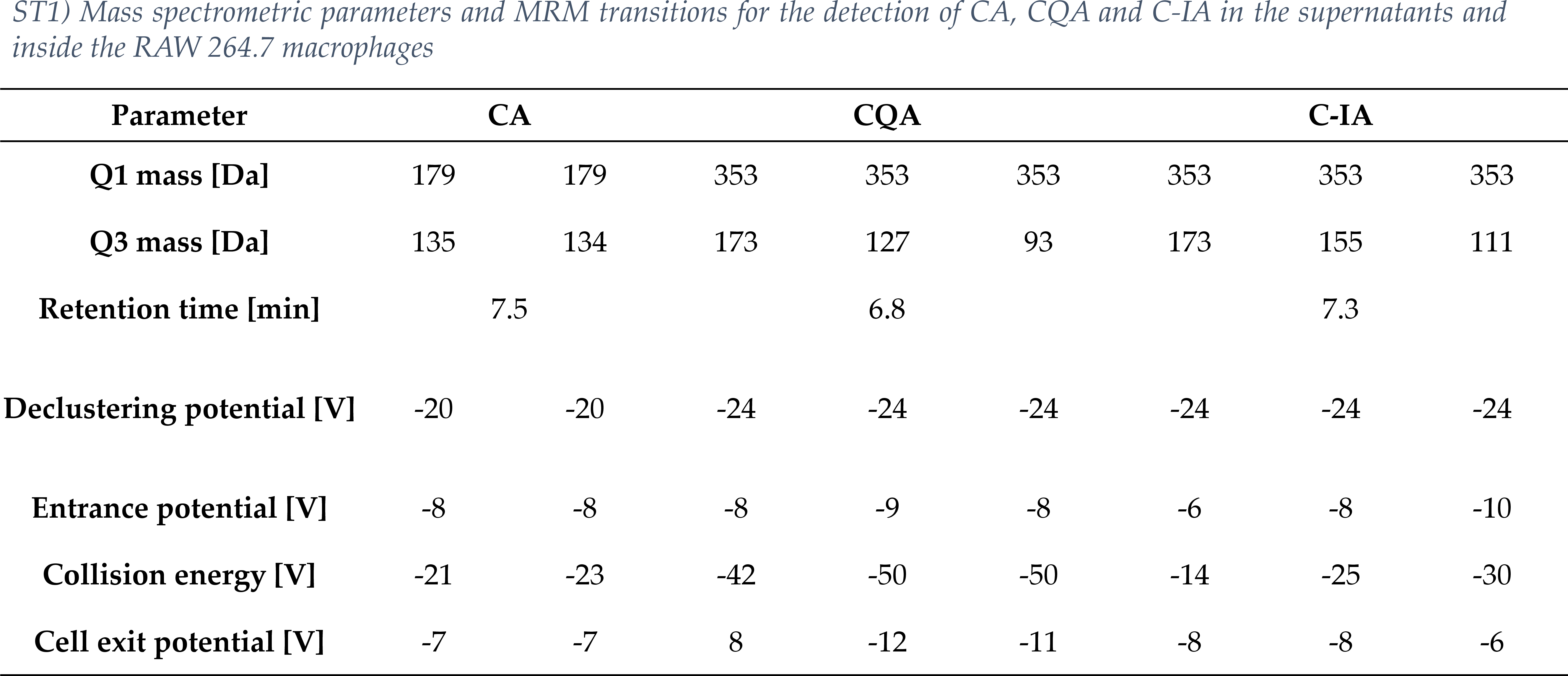

Supplement: Supplementary file 1 [file nutrients-11-00571-s001.zip › Supplemental table 1.tif]

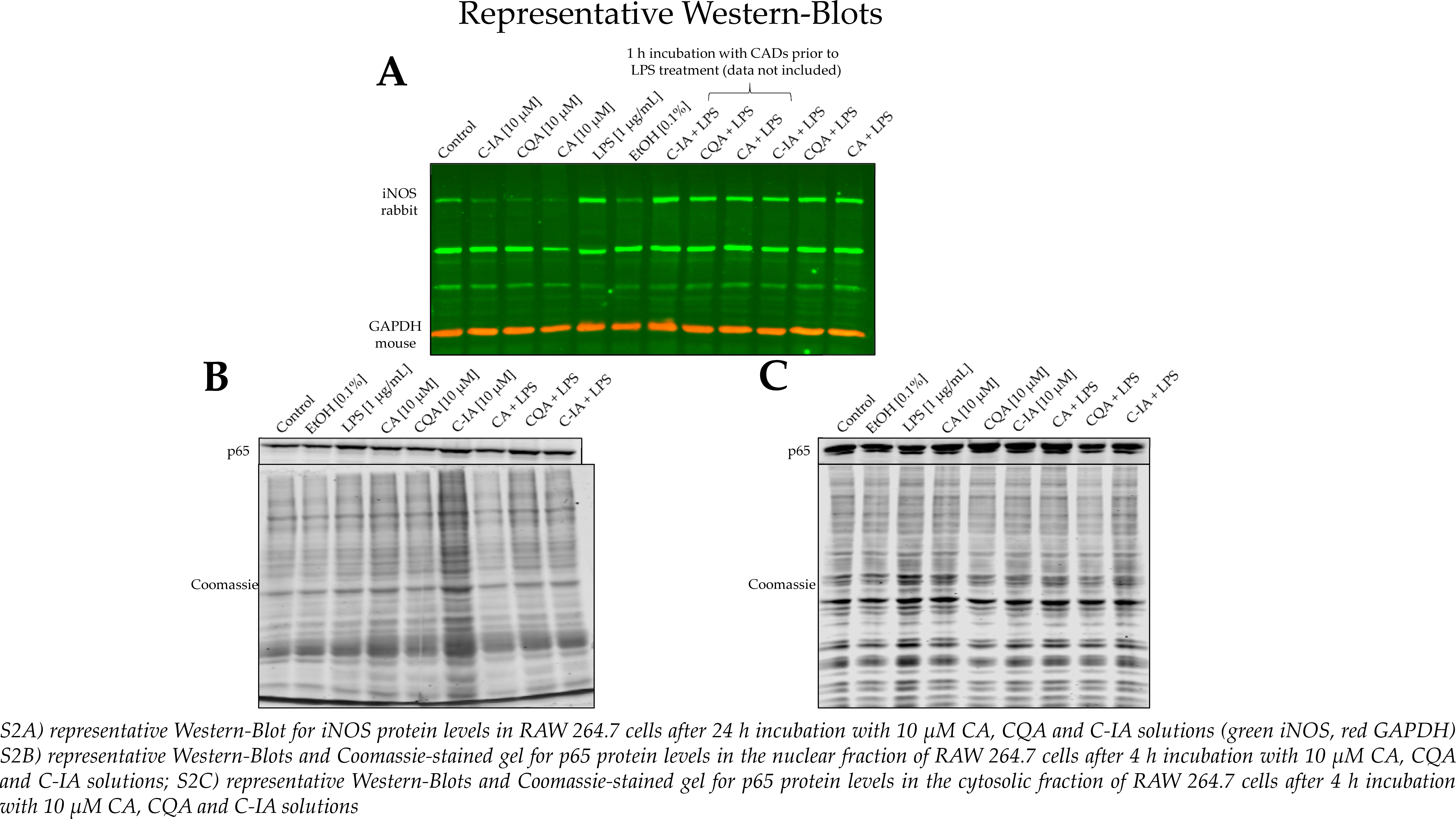

Supplement: Supplementary file 1 [file nutrients-11-00571-s001.zip › Supplemental Figure 2 representative Blots.tif]

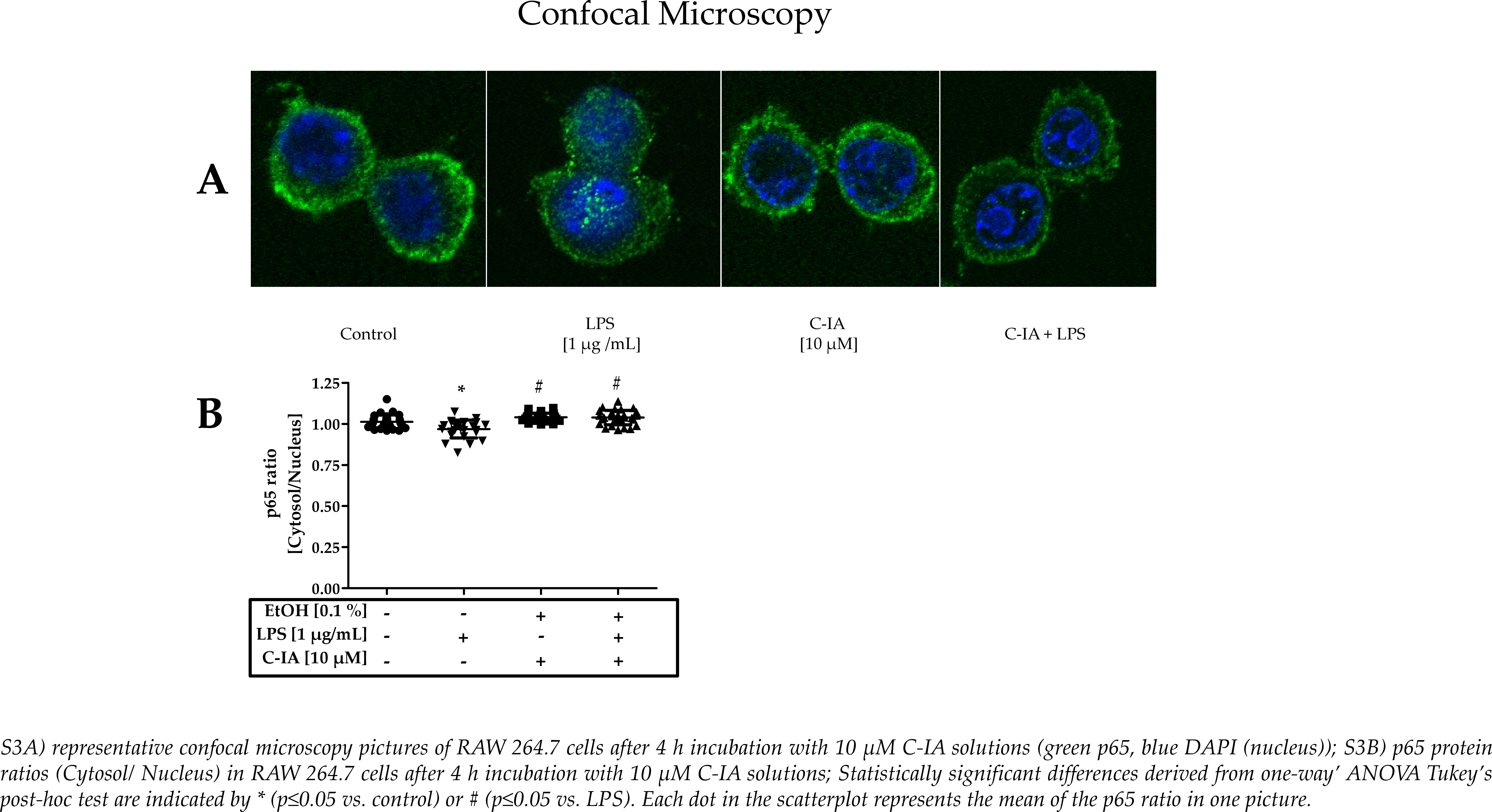

Supplement: Supplementary file 1 [file nutrients-11-00571-s001.zip › Supplemental Figure 3 confocal microscopy.tif]
